# Right atrial function assessed by volume-derived values and speckle tracking echocardiography in patients with hypertrophic cardiomyopathy

**DOI:** 10.1186/s12872-020-01610-1

**Published:** 2020-07-13

**Authors:** Jun Huang, Chao Yang, Cai-Fang Ni, Zi-Ning Yan, Li Fan, Xiang-Ting Song

**Affiliations:** 1 Department of Echocardiography, the Affiliated Changzhou No.2 People’s Hospital with Nanjing Medical University, Changzhou, 213003 China; 2 Department of Interventional Radiology, the First Affiliated Hospital of Soochow University, Suzhou, 215006 China

**Keywords:** Hypertrophic cardiomyopathy, Strain, Strain rate, Volume

## Abstract

**Background:**

To detect the right atrial (RA) functions in hypertrophic cardiomyopathy (HCM) patients by using volume-derived values and two-dimensional strain.

**Methods:**

Thirty-two HCM patients and 34 age and gender matched normal controls were enrolled for this study. RA volume-derived values were measured by using 2D ultrasonic images. RA strain (S-reservoir, S-conduit, S-booster pump) and strain rate (SR-reservoir, SR-conduit, SR-booster pump), representing the reservoir, conduit and booster pump functions, respectively, were measured by EchoPAC.

**Results:**

Total RA emptying fraction (RAEF) and RA expansion index in HCM patients were significantly lower than normal controls (*p* < 0.05). The values of S-reservoir, S-conduit, Sr-reservoir and Sr-conduit in HCM patients were significantly lower than normal controls (*p* < 0.001). Although there were no significant differences in S-booster pump and Sr-booster pump between HCM patients and normal controls, the absolute values in HCM patients were lower than normal controls.

**Conclusions:**

In this study, we concluded that RA dysfunctions, including the reservoir and conduit functions were impaired in HCM patients.

## Background

Hypertrophic cardiomyopathy (HCM) is a genetic disease which is often diagnosed in the clinic. It is characterized by asymmetric left ventricular (LV) hypertrophy and involves the interventricular septum [1, 2]. With the development of imaging technology, such as echocardiography, cardiac radionuclide imaging and magnetic resonance imaging (Cardiac MRI), the discovery and diagnosis of HCM become more and more easy [3–9]. However, Echocardiography is considered one of the most useful technique in screening for HCM patients.

Right atrial (RA) functions play a key role in the cardiac also like left atrial (LA): as a reservoir during right ventricular (RV) systole, a conduit from vena cave veins and coronary sinus to the RV during early diastole and a booster pump in late diastole [10]. Traditional two-dimensional echocardiography detected the RA diameter, containing the middle, longitudinal diameter, however, it is simple and can’t assess RA functions comprehensive.

Recently, two-dimensional speckle tracking echocardiography (2D-STE) for detecting the strain and strain rate in RA functions were mainly focused on patients with pulmonary artery hypertension, acute pulmonary embolism and tricuspid regurgitation severity [11–13]. These studies were necessary to demonstrate that the technique could detect the RA functions flexibility and accuracy.

The mechanism of the association between RA function and HCM is unknown. Biagini et al. [14] found complex genotypes with double or triple mutations in end-stage HCM patients. Progressive diastolic dysfunction and atrial fibrillation may represent adequate substrates for RA remodeling [15]. A progressive “atrial myopathy” has been also suggested in patients with HCM [16].

Doesch C, et al. [17] found that RA and RV involvement could predict atrial fibrillation (AF) in patients with HCM evaluated by cardiac MRI, reduced tricuspid annular plane systolic excursion (TAPSE) and RA dilatation could serve as determinable markers of AF in patients with HCM. McCullough SA, et al. [18] also elevated RA pressure in HCM patients was associated with left-sided heart failure and was an independent predictor of all-cause mortality and new-onset AF. Limongelli G, et al. [15] demonstrated that RA enlargement may serve as a very useful marker of disease progression and adverse outcome in patients with sarcomeric hypertrophic cardiomyopathy. From these previous studies, we conclude that RA dysfunctions detection in HCM patients are very important.

In the study, we combined volume-derived values and two-dimensional strain, strain rate to assess RA functions in HCM patients. To our knowledge, this is the first research to detect the RA functions by volume- and strain-derived values in HCM patients.

## Methods

All HCM patients and normal controls had completed the consent forms for participation in the study. This study was subjected to approval by the ethics committee of “the Affiliated Changzhou No.2 People′s Hospital with Nanjing Medical University”.

### Study samples

Thirty-two HCM patients and 34 age and gender matched normal controls were enrolled for the research. The inclusion criteria for HCM patients were: ① M-mode and 2D echocardiographic evidence of wall thickness ≥ 15 mm in one or more LV myocardial segments and non-dilated LV. ② Absence of another cardiac or systemic disease capable of producing the magnitude of hypertrophy evident in patients with HCM, such as valvular heart disease, hypertension, hyperthyroidism heart disease, uremic cardiomyopathy and coronary heart disease. ③ All enrolled HCM patients were non-obstructive, excluded the apical HCM, there was no obstruction at rest or provocation (peak gradient < 30 mmHg) based on the degree of LV outflow tract obstruction by continuous wave Doppler.

The normal controls had no evidence of hypertension and any other cardiovascular diseases. All of the physical examination, electrocardiogram, and echocardiography were showed normal.

### Two-dimensional doppler echocardiography

All HCM patients and normal controls underwent conventional 2D echocardiography (Vivid E9, GE Healthcare, Horten, Norway), and left atrial diameter (LAd), interventricular septal thickness at the end-diastolic period (IVSd) and LV posterior wall thickness at the end-diastolic period (LVPWd) were measured in the parasternal long-axis view of the LV using M-mode. RV diameter at the middle level (RVd middle), RV diameter at the longitudinal (RVd longitudinal), RV diameter at the base level (RVd basal), RA diameter at the middle level (RAd middle), RA diameter at the longitudinal (RAd longitudinal), and tricuspid annular plane systolic excursion (TAPSE) were measured in the apical four-chamber view. Systolic pulmonary arterial pressure (sPAP) was measured in the parasternal 4-chamber view, if the tricuspid valve regurgitation was absence, sPAP was measured by the regurgitation of pulmonary valve in the parasternal short-axis view at the level of aortic valve or right ventricular outflow view. RV free wall thickness (RVWd) was measured in the subcostal 4-chamber view.

LV end-diastole volume (LVEDV), LV end-systole volume (LVESV) and LV ejection fraction (LVEF) were calculated by bi-plane Simpson′s method.

Peak early and late diastolic velocities of tricuspid valve (E and A, respectively) were measured by pulsed-wave Doppler, and the ratio of E/A was then calculated.

The peak systolic (S′), early (E′) and late (A′) diastolic tricuspid annular velocities were obtained at the RV lateral wall using TDI, and the ratio of E′/A′ was then calculated.

The maximum RA volume (RAVmax), the precontraction RA volume (RAVpre) and the minimum RA volume (RAVmin) were measured in the 2D images using Simpson′s method in the apical four and two chamber views, then total RA emptying volume (RAEV), passive RAEV, active RAEV, total RA emptying fraction (RAEF), passive RAEF, active RAEF and RA expansion index were calculated as following methods: Total RAEV = RAVmax-RAVmin, Passive RAEV = RAVmax-RAVpre, Active RAEV = RAVpre-RAVmin, Total RAEF = Total RAEV / RAVmax * 100, Passive RAEF = Passive RAEV / RAVmax * 100, Active RAEF = Active RAEV / RAVpre * 100, RA expansion index = Total RAEV / RAVmin * 100.

ECG leads were connected to all the participants. The standard high frame rate (40–90 /s) of the apical 4-chamber views (all contained the RA) of three consecutive cycles were stored for off-line analysis.

### Data analysis for RA function

**Fig. 1:**
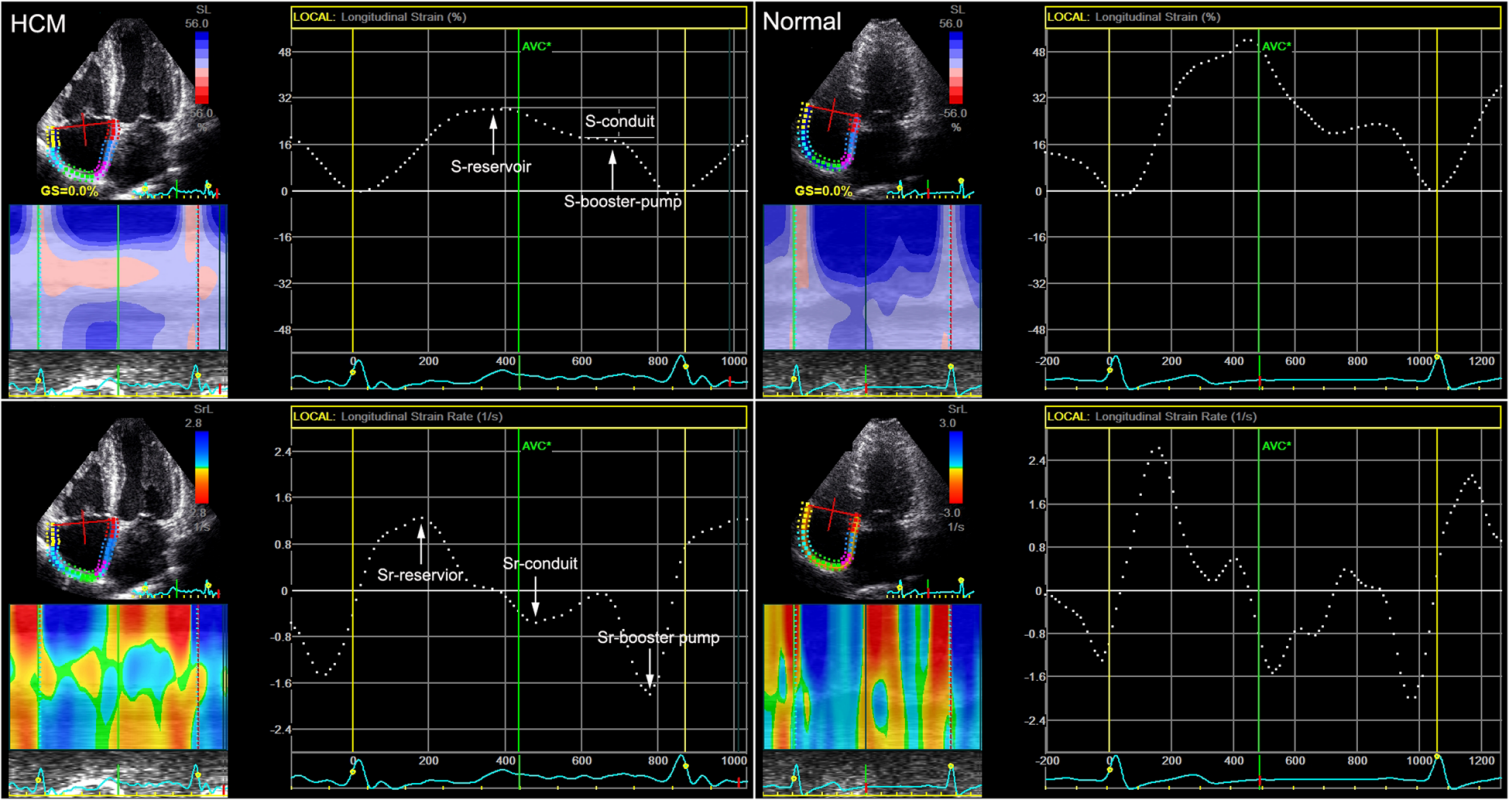
RA functions in apical 4-chamber views in HCM patients and normal controls by using EchoPAC software. S-reservoir, S-conduit and S-booster pump, as well as Sr-reservoir, Sr-conduit and Sr-booster pump, corresponded to the reservoir, conduit and booster pump functions of RA, respectively

The apical 4-chamber views were analysed using the EchoPAC software (EchoPAC Version: 203, GE Vingmed Ultrasound, Norway). Using the button A4C to sketch the endocardial of the RA. The software automatically created a region of interest (ROI) that matched the RA walls. Once the ROI was approved, The RA strain (S-reservoir, S-conduit and S-booster pump) and strain rate (Sr-reservoir, Sr-conduit and Sr-booster pump) were measured (Fig. 1).

### Statistical analysis

All data analyses were performed using SPSS 24.0 software (SPSS, Chicago, IL, USA). Shapiro-Wilk′s test was used to detect the normality of all the values. Differences between HCM patients and normal controls were compared with independent Student′s t-test for the data distribution was normal. For variables with a non-normal distribution, the nonparametric Mann-Whitney test was used. Data are presented as the mean ± standard deviation (mean ± SD). Difference was considered statistically significant in all tests when the *p*-value was < 0.05.

### Intra- and interobserver variability

Twenty patients were randomly selected for intra- and interobserver variability. RA strain and strain rate were measured by two experienced cardiologists who were blinded to patients′ clinical data results. Intraobserver variability was measured at different time. Interobserver variability was determined by repeating measurements from the same off-line images. Intra- and interobserver variability was calculated using intraclass correlation coefficients (ICCs).

## Results

Clinical baseline parameters and 2D echocardiographic characteristics between HCM patients and normal controls were shown in the Table 1.

**Table 1:** Clinical baseline parameters and 2D echocardiographic characteristics between HCM patients and normal subjects (mean ± SD)

Variable	Values		*P* Value
	HCM(32)	Normal(34)	
Clinical baseline parameters			
Age (years)	47 ± 14	46 ± 12	0.779
Male	19 (32)	19 (34)	
Heart Rate (bpm)	70 ± 11	72 ± 12	0.391
NYHA Class			
I	(30)32		
II	(2)32		
III	(0)32		
IV	(0)32		
Medical treatment			
β-Blockers	(27)32		
Calcium channel blockers	(5)32		
Disopyramide	(0)32		
Diuretics	(0)32		
ICD	(0)32		
2D echocardiographic characteristics			
LAd (mm)	42.06 ± 4.91	35.09 ± 3.38	**< 0.001**
IVDd (mm)	19.41 ± 4.09	9.21 ± 1.04	**< 0.001**
LVPWd (mm)	10.19 ± 1.15	9.21 ± 1.04	**0.001**
LVEF(%)	66.59 ± 6.39	65.20 ± 5.30	0.339
RVWd (mm)	2.50 ± 0.51	2.47 ± 0.51	0.815
RVd middle (mm)	29.28 ± 4.26	27.59 ± 3.02	0.066
RVd longitudinal (mm)	52.31 ± 4.40	52.02 ± 5.16	0.812
RVd basal (mm)	25.09 ± 3.44	24.62 ± 3.19	0.562
sPAP (mmHg)	20.50 ± 4.69	18.56 ± 4.44	0.090
RAd middle (mm)	37.09 ± 3.89	37.82 ± 3.83	0.445
RAd longitudinal (mm)	48.93 ± 4.43	46.74 ± 3.42	**0.027**
TAPSE (mm)	21.25 ± 2.06	21.94 ± 1.97	0.169
E	0.48 ± 0.12	0.57 ± 0.09	0.001
A	0.42 ± 0.09	0.40 ± 0.07	0.268
E/A	1.19 ± 0.34	1.47 ± 0.28	0.001
S′(cm/s)	10.41 ± 1.89	9.92 ± 1.42	0.234
E′ (cm/s)	−7.53 ± 2.04	−8.74 ± 1.79	**0.013**
A′ (cm/s)	−11.13 ± 2.20	−10.53 ± 2.37	0.289
E′/A′	0.71 ± 0.24	0.89 ± 0.34	0.016
TR grade			
Absence/Slight	(30)32	(32)34	
Mild	(2)32	(2)34	
Moderate	(0)32	(0)34	
Severe	(0)32	(0)34	

*Abbreviations*: *LAd* left atrial diameter, *IVDd* inter-ventricle septal wall diameter in the diastole period, *LVPWd* left ventricular posterior wall diameter in the diastole period, *LVEF* left ventricular ejection fraction, *RV* right ventricular, *RVd middle* RV diameter at the middle level, *RVWd* RV free wall diameter, *RVd longitudinal* RV diameter at the longitudinal, *RVd basal* RV diameter at the base level, *sPAP* systolic pulmonary arterial pressure, *RA* right atrial, *RAd middle* RA diameter at the middle level, *RAd longitudinal* RA diameter at the longitudinal, *TAPSE* tricuspid annular plane systolic excursion, *E* Peak early velocities of tricuspid valve were measured by pulsed-wave Doppler, *A* Peak late velocities of tricuspid valve were measured by pulsed-wave Doppler, *S′* peak systolic annular velocities obtained at RV lateral wall by using TDI, *E*′ peak early diastolic annular velocities obtained at RV lateral wall by using TDI, *A′* peak late diastolic annular velocities at RV lateral wall by using TDI, *TR* tricuspid valve regurgitation

There were significant differences in LAd, IVSd, LVPWd and RAd longitudinal, E, E/A, E′ and E′/A′ between HCM patients and normal controls (*p* < 0.05). LAd, IVSd, LVPWd and RAd longitudinal were significantly larger than normal controls, while E, E/A, E′ and E′/A′ were significantly lower than normal controls. No significant differences were found in LVEF, RVWd, RVd middle, RVd basal, RAd middle, TAPSE, A, S′ and A′ (*p* > 0.05).

**Fig. 2:**
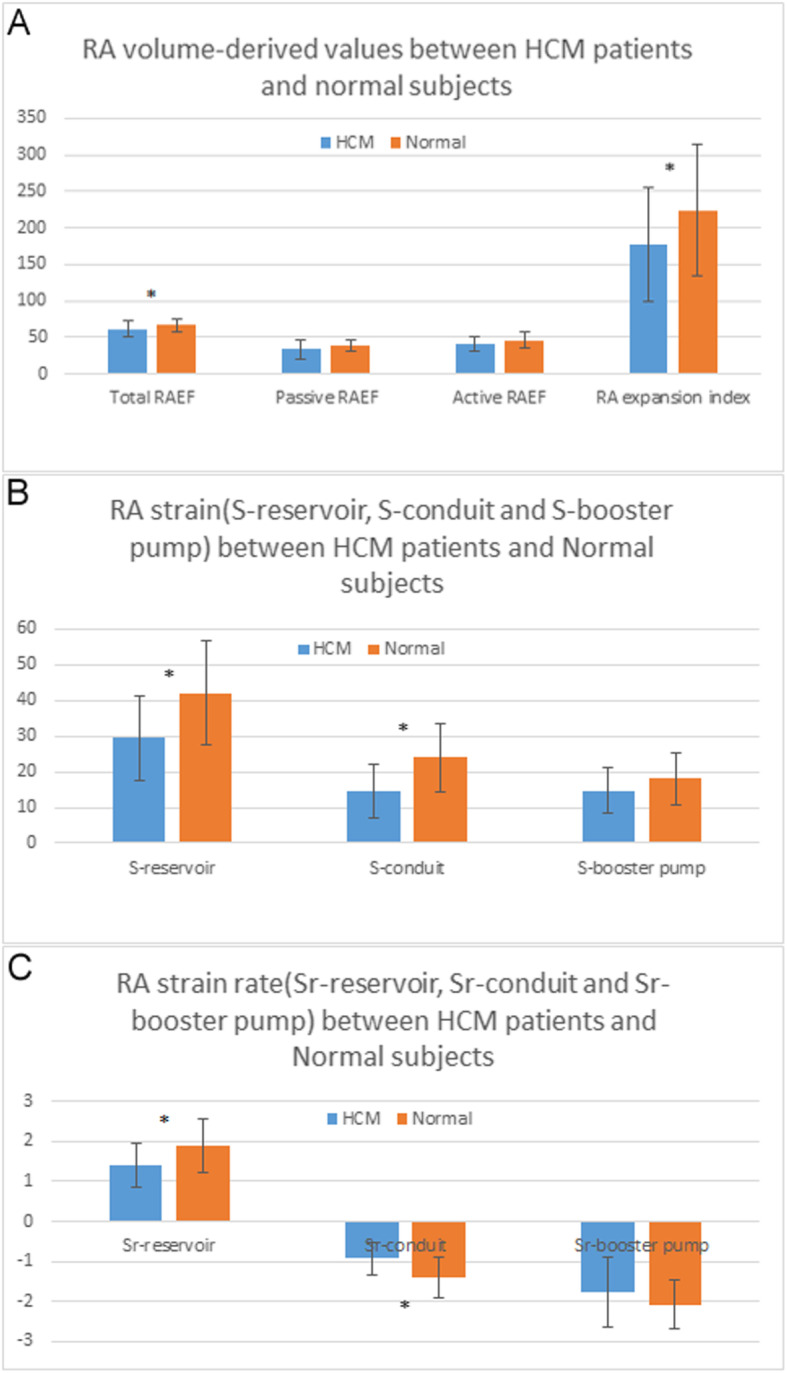
RA volume-derived values (**a**), RA strain (**b**) and strain rate (**c**) between HCM patients and normal controls (* means *P* < 0.05)

2D echocardiographic RA volume and volume-derived values between HCM patients and normal controls were shown in Table 2, Fig. 2a.

**Table 2:** 2D echocardiographic RA volume and function between HCM patients and normal subjects (mean ± SD)

Variable	Values		*P* Value
	HCM(32)	Normal(34)	
RAVmax (ml)	34.19 ± 9.16	36.44 ± 7.70	0.282
RAVpre (ml)	22.94 ± 8.87	22.26 ± 5.01	0.704
RAVmin (ml)	13.66 ± 6.87	12.09 ± 4.11	0.261
Total RAEV (ml)	20.53 ± 5.14	24.35 ± 5.96	**0.007**
Passive RAEV (ml)	11.25 ± 4.54	14.18 ± 4.48	**0.001**
Active RAEV (ml)	9.28 ± 3.52	10.18 ± 3.01	0.270
Total RAEF(%)	61.15 ± 11.04	66.87 ± 9.06	**0.024**
Passive RAEF(%)	33.84 ± 13.06	38.68 ± 8.09	0.073
Active RAEF(%)	41.46 ± 10.31	46.29 ± 11.14	0.075
RA expansion index(%)	178.04 ± 78.18	224.25 ± 90.91	**0.031**

*Abbreviations*: *RAVmax* maximum RA volume, *RAVpre* precontraction RA volume, *RAVmin* minimum RA volume; *Total RAEV* total RA emptying volume, *Active RAEV* active RA emptying volume, *Passive RAEV* passive RA emptying volume; *Total RAEF* total RA emptying fraction; *Active RAEF* active RA emptying fraction, *Passive RAEF* passive RA emptying fraction

There were significant differences in Total RASV, Passive RASV, Total RAEF and RA expansion index (*p* < 0.05), and all the values were significantly lower than normal controls. Although there were no significant differences in RAVmax, RAVpre, RAVmin, Active RASV, Passive RASV and Active RAEF (*p* > 0.05), the absolute values of Active RASV, Passive RASV and Active RAEF were lower than normal controls.

RA reservoir, conduit and booster pump functions between HCM patients and normal controls by detecting the RA Strain and strain rate were shown in Table 3, Fig. 2b, c

**Table 3:** Comparison of the RA reservoir, conduit and booster pump function between HCM patients and normal subjects by detecting the RA Strain and strain rate (mean ± SD)

Variable	Values		*P* Value
	HCM(32)	Normal(34)	
S-reservoir(%)	29.42 ± 12.02	41.99 ± 14.58	**< 0.001**
S-conduit(%)	14.70 ± 7.61	23.95 ± 9.69	**< 0.001**
S-booster pump(%)	14.72 ± 6.45	18.04 ± 7.13	0.052
Sr-reservoir(S^−1^)	1.40 ± 0.56	1.90 ± 0.67	**< 0.001**
Sr-conduit(S^− 1^)	−0.93 ± 0.41	−1.43 ± 0.51	**< 0.001**
Sr-booster pump(S^−1^)	− 1.77 ± 0.88	−2.07 ± 0.61	0.104

*Abbreviations*: *S-reservoir* RA strain corresponding to reservoir function, *S-conduit* RA strain corresponding to conduit function, *S-booster pump* RA strain corresponding to booster pump function, *SR-reservoir* RA strain rate corresponding to reservoir function, *SR-conduit* RA strain rate corresponding to conduit function, *SR-booster pump* RA strain rate corresponding to booster pump function

There were significant differences in S-reservoir, S-conduit, Sr-reservoir and Sr-conduit between HCM patients and normal controls (*p* < 0.001). All the values were significantly lower than normal controls. Although there were no significant differences in S-booster pump and Sr-booster pump between HCM patients and normal controls, the absolute values in HCM patients were lower than normal controls.

### Intra- and interobserver variability

The results for the intraobserver and interobserver variability for the RA strain and strain rate upon repeated measurements in 20 random patients were shown in Table 4. ICC values were higher for intraobserver and interobserver variability when the same images were analyzed by the two different cardiologists. What′s more, the results also demonstrated that the analysis of the software had the well repeatability and reliability in this study.

**Table 4:** ICCs for intra- and interobserver variability for RA strain and strain rate

Variable	Interobserver variability		Intraobserver variability	
	ICC	95% CI	ICC	95% CI
S-reservoir (%)	0.996	0.989–0.998	0.997	0.992–0.999
S-conduit (%)	0.985	0.962–0.994	0.989	0.971–0.996
S-booster pump (%)	0.981	0.951–0.992	0.992	0.979–0.997
Sr-reservoir (S^−1^)	0.984	0.960–0.994	0.986	0.966–0.995
Sr-conduit (S^−1^)	0.984	0.960–0.994	0.971	0.925–0.988
Sr-booster pump (S^−1^)	0.991	0.979–0.997	0.983	0.958–0.993

## Discussion

The main findings of the research were: RA reservoir and conduit functions were impaired in HCM patients when compared with normal controls.

2D-STE is a new echocardiographic technique that has been validated for the assessment of LV, LA, RV and RA functions in HCM patients by previous studies [19]. Also, our group had been done some researches in LA and LV function of HCM. Huang J, et al. [20, 21] detected the longitudinal and circumferential strain of different myocardium layers, radial strain, and longitudinal rotation by using 2D-STE, and demonstrated that the long-axis and short-axis LV functions in HCM patients were impaired. Huang J, et al. [22] also detected the LA dysfunction in HCM patients by 2D-STE and volume-derived values. Fujimoto K et al. [23] elucidated the impact of LA function by 2D-STE and cardiac MRI, and found loss of LA active function was associated with increased cardiac events in patients with HCM.

RA functions play an important role in the cardiac and 2D-STE is a feasible, angle-independent method for the evaluation of RA function [24]. Researchers mainly focused RA functions on pulmonary artery hypertension, acute pulmonary embolism, hypertension, the influence of severity tricuspid regurgitation, right ventricular myocardial infarction, tetralogy of Fallot, and so on [11, 12, 25–27]. However, till now, we have not found a research about RA functions in HCM patients by using 2D-STE and RA volume-derived values.

Recently, some researchers have found RV functions were impaired in HCM patients by 2D-STE. Cincin A, et al. [28] explored RV mechanical function in HCM patients by demonstrated 2D-STE-derived RV systolic function was impaired in HCM patients when compared with healthy subjects. D’Andrea A, et al. [29] analyzed RV systolic function in patients with HCM at rest and during exercise, and found that RV contractile reserve for HCM was impaired. This study demonstrates for the first time that 2D-STE algorithm applied to routine grey-scale 2D images represents a promising and feasible non-invasive technique to assess RA functions in HCM patients.

From basic 2D echocardiographic characteristics between HCM patients and normal controls, we found RA and LA diameters were dilated in HCM patients. From the volume-derived values, total RAEV, total RAEF and RA expansion index represented the reservoir function, passive RAEV represented the conduit function were significantly lower than normal subjects, the results indicated that RA reservoir and conduit functions were impaired. Strain and strain rate analysis also demonstrated that reservoir and conduit functions were impaired. Although the booster pump function of volume and strain derived-values had no significant differences between HCM patients and normal controls, the absolute values in HCM patients were lower than normal controls. HCM is a progressive disease characterized by LV hypertrophy and fibrosis. LA, LV and RV dysfunctions were observed and demonstrated that the functions were damaged. Our findings may stress the significance of the evaluation of RA dysfunction because it permits us to evaluate RV function on the other hand.

## Conclusion

From the study, we concluded that RA dysfunctions, including the reservoir and conduit functions were impaired in HCM patients.

## Data Availability

The datasets used and/or analysed during the current study available from the corresponding author on reasonable request.

## Abbreviations

HCM: Hypertrophic cardiomyopathy
2D-STE: Two-dimensional speckle tracking echocardiography
MRI: Magnetic resonance imaging
RA: Right atrial
RV: Right ventricular
LA: Left atrial
LV: Left ventricular
LAd: Left atrial diameter
IVSd: Interventricular septal thickness at the end-diastolic period
LVPWd: LV posterior wall thickness at the end-diastolic period
RVWd: RV free wall diameter
RVd middle: RV diameter at the middle level
RVd longitudinal: RV diameter at the longitudinal
RVd basal: RV diameter at the base level
RAd middle: RA diameter at the middle level
RAd longitudinal: RA diameter at the longitudinal
TAPSE: Tricuspid annular plane systolic excursion
sPAP: Systolic pulmonary arterial pressure
LVEDV: LV end-diastole volume
LVESV: LV end-systole volume
LVEF: LV ejection fraction
RAEV: RA emptying volume
RAEF: RA emptying fraction
ROI: Region of interest
ICCs: Intraclass correlation coefficients
